# The effects of whole milk compared to skim milk and apple juice consumption in breakfast on appetite and energy intake in obese children: a three-way randomized crossover clinical trial

**DOI:** 10.1186/s40795-018-0253-8

**Published:** 2018-12-10

**Authors:** Shima Kavezade, Hassan Mozaffari-Khosravi, Majid Aflatoonian, Mehdi Asemi, Sanaz Mehrabani, Amin Salehi-Abargouei

**Affiliations:** 1 Nutrition and Food Security Research Center, Shahid Sadoughi University of Medical Sciences, Yazd, Iran; 2 Department of Nutrition, School of Public Health, Shahid Sadoughi University of Medical Sciences, Yazd, PO Code 8915173160 Iran; 3 Diabetes Research Center, Shahid Sadoughi University of Medical Sciences, Yazd, Iran; 4 Department of Pediatrics, Faculty of Medicine, Shahid Sadoughi University of Medical Sciences, Yazd, Iran; 5 Shahrekord branch, Islamic Azad University, Shahrekord, Iran; 6 Food Security Research Center, Isfahan University of Medical Sciences, Isfahan, Iran; 7 Department of clinical Nutrition, School of Nutrition and Food Science, Isfahan University of Medical Sciences, Isfahan, Iran

**Keywords:** Full-fat milk, Skim milk; dairy fat; appetite, Energy intake, Child

## Abstract

**Background:**

A limited number of studies have examined the effect of dairy on satiety and short-term energy intake among children; furthermore we are not aware of any study comparing high and low-fat dairy products regarding their effect on appetite and short-term energy intake. Our objective was to assess the effect skim milk (SM) compared to whole milk (WM) and apple juice (AJ) on satiety and energy intake at lunch among 10–12 y children with obesity.

**Methods:**

Fifty children with obesity who aged 10–12 y were randomized to consume a fixed content breakfast with 240 ml of SM, AJ, or WM for two consecutive days. The study was a three-way randomized crossover study; therefore each participant served as his/her own control. The total appetite, hunger, fullness, desire to eat and prospective consumption were measured using a visual analogue scale (VAS) before breakfast and every one hour after breakfast until a freely consumed lunch. VAS scores and energy intakes were compared using repeated measures procedure.

**Results:**

Forty-eight participants (24 girls and 24 boys) completed the study. The energy intake was not different between SM, AJ and WM periods (adjusted mean ± standard error (SE) of energy intake: SM = 831.27 ± 30.64 Kcal, AJ = 794.92 ± 28.72 Kcal, WM = 798.87 ± 24.09 Kcal; *P* = 0.56). The effect was the same for either gender. Children reported higher satiety score 4 h after drinking WM with breakfast compared with SM (*P* < 0.05). The same association was found only in girls. Furthermore, SM significantly reduced appetite compared to AJ, 2 h after preloads in girls (*P* < 0.05).

**Conclusions:**

Full-fat milk may have favorable effects on satiety but not energy intake in subsequent meal compared to skim milk among the children with obesity. Future studies with longer follow-up periods are needed to confirm these results.

**Trial registration:**

The study protocol was registered with the Iranian registry of clinical trials on 9th October 2016 (registration ID: IRCT2016072012571N5).

**Electronic supplementary material:**

The online version of this article (10.1186/s40795-018-0253-8) contains supplementary material, which is available to authorized users.

## Background

Obesity has become a global concern especially among children [1]. According to the reports provided by world health organization (WHO) in 2010, it is assumed that around 43 million children experienced obesity and more than twice this population were exposed to overweight all around the world [1]. Kelishadi et al. showed that obesity is affecting 8.8% of Iranian children based on the criteria explained by Center for Disease Control (CDC) [2]. Childhood obesity is of great importance because several investigations have provided evidence that there is a direct relationship between childhood obesity and chronic non-communicable diseases such as diabetes mellitus, cardiovascular diseases, and depression in later life [3].

Several strategies have been introduced for controlling the appetite, and energy intake for the management of body weight [4]. In particular, the approaches to control satiety and energy intake are important for children with obesity, as the low-calorie diets are not recommended for this age group because of their possible adverse effect on growth [5]. Although the studies have led to inconsistent results, a number of publications have suggested that milk and dairy products intake may reduce appetite and short-term energy intake [6]. For instance, a study done by Mehrabani et al. on 34 boys with obesity showed that drinking low-fat milk causes a significant decrease in appetite and energy intake at launch when compared with water and apple juice as the iso-volumic and iso-volumic/iso-energetic control beverages, respectively [7]. On the other hand, another study showed that increasing the dairy consumption from one portion per day to 3 does not affect appetite and may lead to increased energy intake and consequently weight gain [8]. In another study, the effect of receiving orange juice, 1% milk fat and cola in comparison with water on hunger, satiety and energy intake in the next meal was studied. All of these beverages increased the satiety and reduced starvation rates compared to water [9].

The effect of dairy on satiety is attributed to several components such as protein, lactose, and lipids including linoleic acid and conjugated fatty acids. However, it is not clear that which component is responsible for the effect [10]. Researchers have suggested that proteins might play a strong role in controlling appetite. The effect of proteins in the short term regulation of the food intake is related to the increased plasma concentration of peptide hormones in the gastrointestinal tract, which reduces gastric emptying, gastrointestinal movements and appetite [10]. Furthermore, the fat content of dairy is also proposed to have a role in reducing appetite [10]. The mechanism through which the fat compartment of dairy affects satiety is not well understood; however, studies have shown that the type and the structure of fatty acids, their chain length, and degree of saturation play an essential role in appetite [11]. It has been shown that fat can prolong the passage of food from the gastrointestinal tract and stimulate the release of peptide YY (PYY), cholecystokinin, glucagon-like peptides, and suppresses appetite and energy intake [12].

While studies have come to controversial results regarding the effect of dairy consumption on satiety in children, we are not aware of any study trying to examine the appetite lowering effect of dairy fat. Therefore, in the present study, we aimed to assess the effect of consuming skim milk compared to full-fat milk and apple juice in breakfast on appetite and short-term energy intake in children with obesity through a three-way randomized cross-over clinical trial. We compared the skim milk with the full-fat milk to see the possible effect of fat content of dairy on appetite and energy intake at lunch. Apple juice was also selected as the control beverage to see if the skim milk lowers the satiety when compared to an iso-volumic/iso-energetic beverage.

## Methods

### Study protocol

This was a three-way repeated measurement randomized, controlled cross-over trial in which each individual served as his/her own control. The study protocol was approved by the ethics committee of the School of Public Health, Shahid Sadoughi University of Medical Sciences (Identification number: IR.SSU.SPH.REC.1394.104) and was registered on 9th October 2016 in the Iranian registry of clinical trials (IRCT, www.irct.ir, registration ID: IRCT2016072012571N5; URL: http://en.irct.ir/trial/12621). The purpose of the study was explained to all parents of the children participating in the study and to their school authorities (principles, assistants, and teachers), and written consents were obtained from the parents.

### Randomization and blinding

**Fig. 1:**
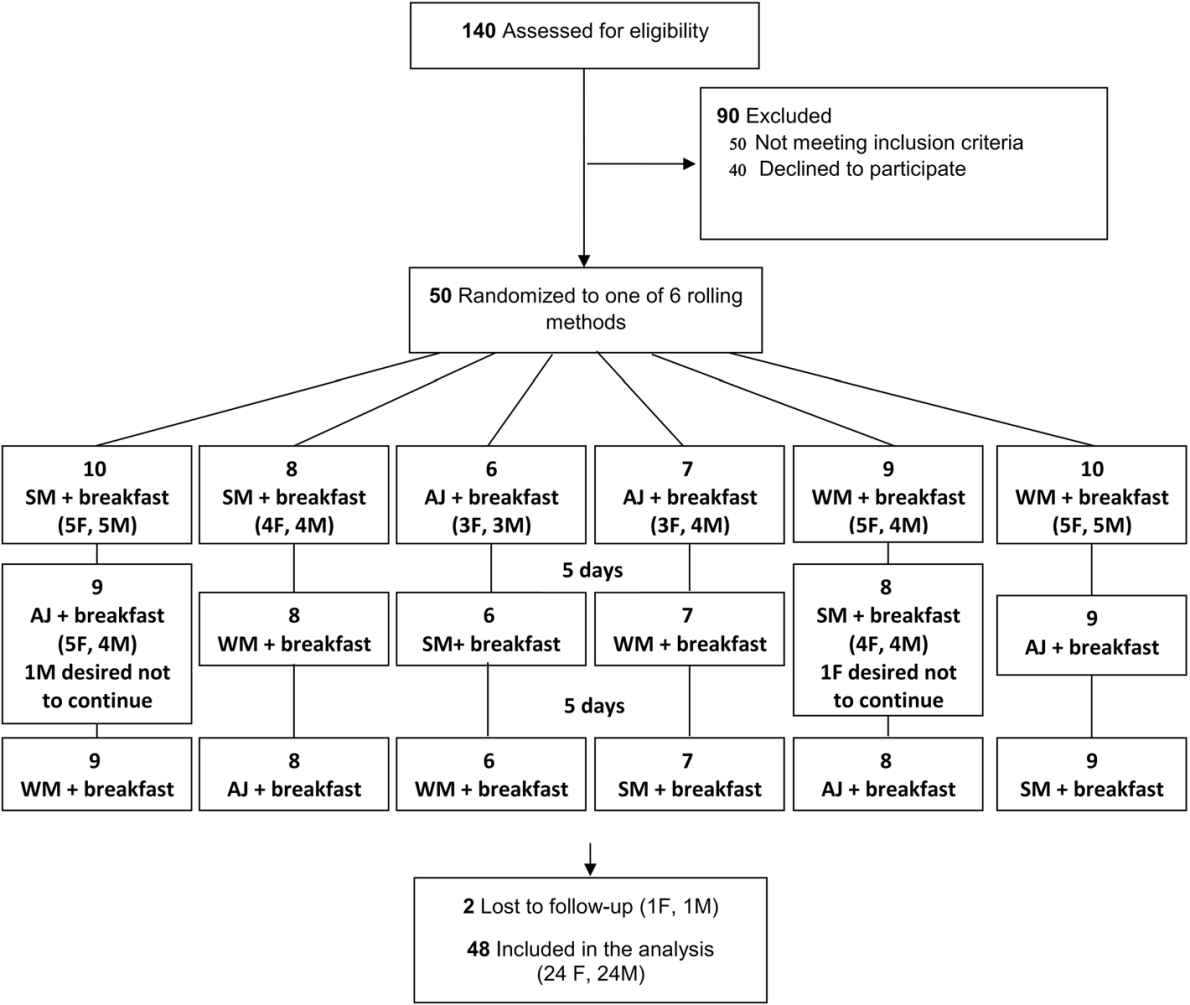
The study flow diagram. Each intervention was conducted for two consecutive days (Saturdays and Sundays). SM: skim milk, AJ: apple juice, WM: whole milk, F: females and M: males

The present study tried to examine the effect of three intervention drinks [skim milk:SM (0% fat), whole milk:WM [3% fat], and apple juice:AJ] on satiety and short-term energy intake in the context of a three-way randomized cross-over trial. Therefore, participants were randomly assigned into 6 rolling methods (SM-WM-AJ, SM-AJ-WM, WM-SM-AJ, WM-AJ-SM, AJ-WM-SM, and AJ-SM-WM) to receive the three iso-volumic drinks along with a fixed energy breakfast (Fig. 1). The randomization was done using statistical package for social sciences (SPSS) software. Intervention periods lasted for 2 consecutive days, and there were 5 days of wash-out period between the interventions. We selected two consecutive days because our previous experience showed that two days of intervention is enough to show the effect of low-fat milk intake on satiety and short-term energy intake [7]. The whole and the skim milk were coded by a researcher out of the study and provided for the study personnel. Therefore the personnel and the participants were blinded to the type of milks. However it was not possible for the researchers to blind the participants to the apple juice.

### Eligibility criteria

Participants were selected from two elementary schools (one for girls and one for boys) Isfahan, Iran. Children aged 10–12 years old who were with obesity based on the reference curves provided by WHO (obesity was defined as having a body mass index (BMI) above the 95th percentile based on WHO BMI-for-age charts), were not on a special diet or had no intention to go on diet during the study period, did not have intolerance to cow’s milk, did not report any history of congenital or metabolic diseases using information provided from parents, and regularly consume breakfast were included in the present study. It was planned to exclude the participants if they denied participating in at least two of the intervention periods.

### Intervention details

On the intervention days, boys and girls attended school, while they were in the fasted state at 7 a.m. Subjects were given breakfast meal that was composed of 65 g of an Iranian whole-wheat bread (called sangak), 15 g of low-fat cheese and 12 g of walnut with one of the three test beverages: skim milk, apple juice or whole milk (full-fat) milk. The food and nutrient content of the breakfast along with the test beverages are provided in Additional file 1: Table S1. Subjects were checked to completely eat their breakfast till 7:30 a.m. After breakfast, the study attendants were prohibited to eat and drink anything except water and a small portion of fruit (apple) that contained approximately 15 g of carbohydrates, until lunchtime. A lunch meal was provided at 12:30 for all of the participants in the schools’ hall. The food items provided for lunch were the same for all participants and intervention periods. The food items which were provided for the first day of each intervention period were cooked rice, kebab, yogurt, cola beverage, pasta and bottled water and the food items provided for the second intervention days were cooked rice, chicken, yogurt, bottled water and cola beverage. Subjects were allowed to freely select their food. Participants were asked to continue to eat until they felt absolutely full. The food items were provided in packages and each food item was weighed and then included in its package. Participants were allowed to select as much as packages they want. After the participants finishing their lunch, the remaining foods in each package were weighted.

### Anthropometric measurements

A wall-mounted stadiometer was used to measure the height, and the weight was assessed using a digital scale, whereas subjects were wearing minimal clothing. BMI was computed as weight (kg) divided by the square of height (m^2^). Anthropometric measurements were performed three times for each person and the value that was shown at least twice for each subject was recorded.

### Assessment of the appetite

The participants filled a visual analogue scale (VAS) which was previously used in children [13] before consumption of the breakfast (T0), right after breakfast consumption (T1) and each hour after breakfast until before lunch [8:30 a.m. (T2), 9:30 a.m. (T3), 10:30 a.m. (T4), 11:30 a.m. (T5), 12:30 a.m. (T6)]. Participants were trained by the study members on how to complete the questionnaire. Each VAS consisted of 4 questions: “How hungry do you feel at this moment?”, “How full do you feel at this moment?”, “How strong is your desire to eat at this moment?” and “How much food do you think you could eat at this moment?” which assessed the components of appetite including the desire to eat, fullness, hunger, and prospective consumption respectively. The answers to these questions were made through placing a cross on the 100-mm lines anchored on their end with the most positive and the most negative answers for each question (I do not feel hungry at all/I feel very hungry, I do not feel satiety at all/I feel very satiety, I have no desire/I so desire, no/very much). The scores for each of these questions would be from 0 to 100; therefore, the total score for appetite ranged from 0 to 400.

### Assessment of the energy intake and the physical activity

Subjects were asked to continue their habitual diet and physical activity during the study period. The dietary intakes consumed by participants on intervention days from breakfast till lunch was measured by weighted dietary record done by an educated nutritionist. Parents were also asked to complete food records for 2 days before, during and 2 days after the intervention days. We converted the food items to grams using household measures and then converted them to energy using nutritionist IV software (version 3.5.2, Axxya Systems, Redmond, Washington, USA). Parents were also asked to record their children’s physical activity 2 days before the intervention, during the intervention days and 2 days after the intervention using predefined forms. All activities and their duration were recorded by parents during the mentioned periods. The recorded activities were converted to metabolic equivalent-hour/day (Met-h/day) using the MET intensity of each activity [14]. We tried to compare the energy intake and physical activity before the three intervention periods because the physical activity and the energy intake before the intervention days may affect the satiety and the energy intake on the intervention days [15, 16].

### Statistical analysis

Descriptive statistics are presented as means ± Standard deviations (SDs) or standard error of means (SEs) where provided. Normality of the outcome variables was assessed using Kolmogorov-Smirnov test. Differences in 1) mean energy intakes at lunch and 2) mean appetite scores, were compared using generalized linear model (GLM) repeated measures procedure considering the test beverages and testing occasions as the repeated factors. Participants’ rolling method, age, and BMI were controlled in different models as between-subject variables. Gender was also considered as a between subjects factor in the analyses for total population. All analyses were done for the total population as well as either gender. *P* values less than 0.05 (2-tailed) were considered as statistically significant. The randomization and the statistical analyses were conducted by the use of statistical package for social sciences software version 20 (IBM SPSS, Tokyo, Japan).

## Results

About 140 children were initially assessed for inclusion criteria. Fifty children did not meet the criteria and the parents of 40 parents declined the invitation for the first meeting which was arranged for describing the objectives of the study. Parents of 50 children gave the informed consent and their children entered into the study. However, one boy and one girl refused to continue the study after the first study period. Therefore, 48 children (24 girls and 24 boys were included in the present study) completed the study and included in the analyses. The study flow diagram is shown in Fig. 1.

The participants were aged 10.77 ± 0.99 years (10.91 ± 0.92 for girls and 10.62 ± 1.05 for boys) and there was no significant difference between genders in terms of their age (*P* = 0.31). The general characteristics of the study participants included in the study are presented in Table 1. There was no difference in weight and height between girls and boys (*P* > 0.05); however, the BMI was significantly higher in boys compared to girls (*P* < 0.05).

**Table 1:** The general characteristics of the study participants^a^

	Boys (*n* = 24)	Girls (*n* = 24)	Total population (*n* = 48)	*P* value
Age (y)	10.62 ± 1.05	10.91 ± 0.92	10.77 ± 0.99	0.31
Weight (kg)	57.20 ± 11.28	52.20 ± 8.58	54.70 ± 10.23	0.09
Height (m)	1.46 ± 0.11	1.48 ± 0.06	1.47 ± 0.09	0.59
BMI (Kg/m2)	26.25 ± 2.50	23.68 ± 3.17	24.96 ± 3.11	0.003

^a^Values are reported as mean ± standard deviation (SD). Analyses were done using independent samples t-test

The crude and multivariable-adjusted meanb± standard error (SE) for energy intake and physical activity level (Met-min/day) two days before each intervention period in the total population as well as each sex is presented in Table 2. The analysis revealed that the energy intake as well as the physical activity before the intervention periods were not significantly different between skim milk, whole milk and apple juice periods either in crude and multivariable-adjusted models (*P* > 0.05) in the total population. This finding was also true in either gender.

**Table 2:** Total energy intake and Physical activity level before the intervention days^a^

	Boys				Girls				Total population			
	Skim milk	Apple juice	Whole milk	*P*	Skim milk	Apple juice	Whole milk	*P*	Skim milk	Apple juice	Whole milk	*P*
Energy intake (Kcal/day)												
Crude	2073.04 ± 97.72	1782.45 ± 93.43	1740.45 ± 96.13	0.07	1686.36 ± 100.89	1766.12 ± 120.30	1659.49 ± 102.86	0.70	1879.70 ± 74.98	1774.29 ± 75.35	1699.97 ± 69.89	0.16
Model I^b^	2073.04 ± 98.78	1782.45 ± 97.03	1740.45 ± 97.53	0.98	1686.36 ± 103.22	1766.12 ± 125.65	1659.49 ± 106.97	0.71	1879.70 ± 76.46	1774.29 ± 76.90	1699.97 ± 70.47	0.74
Model II^c^	2073.04 ± 99.81	1782.45 ± 97.10	1740.45 ± 95.19	0.99	1686.36 ± 105.75	1766.12 ± 125.16	1659.49 ± 102.15	0.49	1879.70 ± 77.20	1774.29 ± 75.64	1699.97 ± 67.12	0.72
Physical activity (Met-min/day)												
Crude	1387.48 ± 87.50	1434.64 ± 99.19	1490.55 ± 68.24	0.62	1644.40 ± 73.05	1548.57 ± 68.43	1668.37 ± 79.60	0.16	1526.35 ± 59.52	1496.23 ± 58.60	1586.67 ± 54.56	0.31
Model I^b^	1387.48 ± 90.94	1434.64 ± 104.51	1490.55 ± 55.69	0.08	1644.40 ± 73.54	1548.57 ± 70.82	1668.37 ± 77.60	0.14	1526.35 ± 57.45	1496.23 ± 60.24	1586.67 ± 54.99	0.04
Model II^c^	1387.48 ± 94.35	1434.64 ± 108.08	1490.55 ± 52.52	0.08	1644.40 ± 72.27	1548.57 ± 67.11	1668.37 ± 79.13	0.14	1526.35 ± 57.46	1496.23 ± 60.57	1586.67 ± 55.80	0.05

^a^Values are reported as mean ± standard error (SE). The generalized linear model (GLM) repeated measures procedure was done for all analyses

^b^The model was adjusted for age and body mass index (BMI). Gender was also considered as a between subjects factor in the analyses for total population

^c^The model was adjusted for age, body mass index (BMI) and the rolling method

### The effect of test beverages on the energy intake at lunch

The mean ± standard error (SE) for energy intake at lunch based on each intervention period is represented in Table 3. The analysis revealed that the energy intake at lunch was not significantly different between skim milk, whole milk and apple juice periods in girls, boys and total population (P > 0.05). The adjustment for gender, age, BMI and the rolling method did not change the association.

**Table 3:** The energy intake by the study participants at lunch^a^

	Boys				Girls				Total population			
	Skim milk	Apple juice	Whole milk	*P*	Skim milk	Apple juice	Whole milk	*P*	Skim milk	Apple juice	Whole milk	*P*
Energy intake at lunch (Kcal)												
Crude	883.56 ± 38.81	785.46 ± 41.68	855.76 ± 40.26	0.25	778.99 ± 47.28	804.39 ± 39.00	741.99 ± 25.95	0.22	831.27 ± 31.20	794.92 ± 28.28	798.87 ± 25.10	0.60
Model I^b^	883.56 ± 40.26	785.46 ± 42.90	855.76 ± 39.60	0.41	778.99 ± 49.41	804.39 ± 40.77	741.99 ± 25.43	0.94	831.27 ± 31.60	794.92 ± 28.74	798.87 ± 24.25	0.54
Model II^c^	883.56 ± 38.58	785.46 ± 42.61	855.76 ± 39.54	0.41	778.99 ± 48.39	804.39 ± 41.75	741.99 ± 26.01	0.88	831.27 ± 30.64	794.92 ± 28.72	798.87 ± 24.09	0.56

^a^Values are reported as mean ± standard error (SE). The generalized linear model (GLM) repeated measures procedure was done for all analyses

^b^The model was adjusted for age and body mass index (BMI). Gender was also considered as a between subjects factor in the analyses for total population

^c^The association was adjusted for age, body mass index (BMI) and the rolling method

### The effect of beverages on appetite

**Fig. 2:**
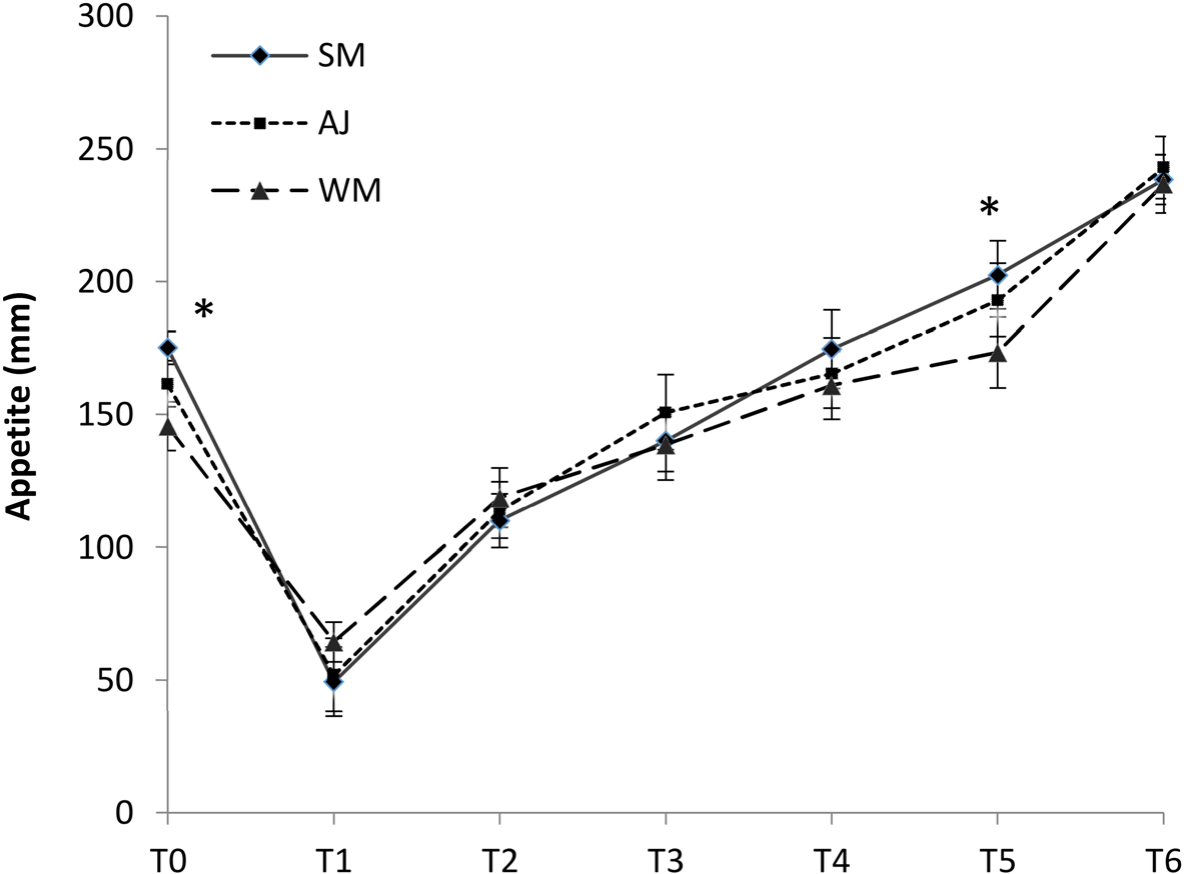
Mean ± SE subjective visual analogue scale (VAS) values for the overall appetite after intake of skim milk (●), apple juice (■) and whole milk (▲) adjusted for age,gender, BMI, and rolling method. Significant differences between the intervention periods are shown with asterisks. Standard errors are appeared by vertical bars

All the analyses for the comparison of mean appetite and its components scores were adjusted for gender, age, BMI, and rolling method. The total appetite score and its components were significantly changed by time for all intervention beverages in all participants as well as either gender (*P* < 0.05). The analysis of the total appetite scores in the total population revealed that the appetite was significantly lower in the whole milk period compared to skim milk before eating the breakfast (*P* < 0.05). Furthermore, the mean satiety level was significantly higher in the whole milk period compared to the skim milk period at 11:30 a.m. (*P* < 0.05). The intervention beverages were not statistically different regarding their effect on appetite in other testing occasions (Fig. 2).

**Fig. 3:**
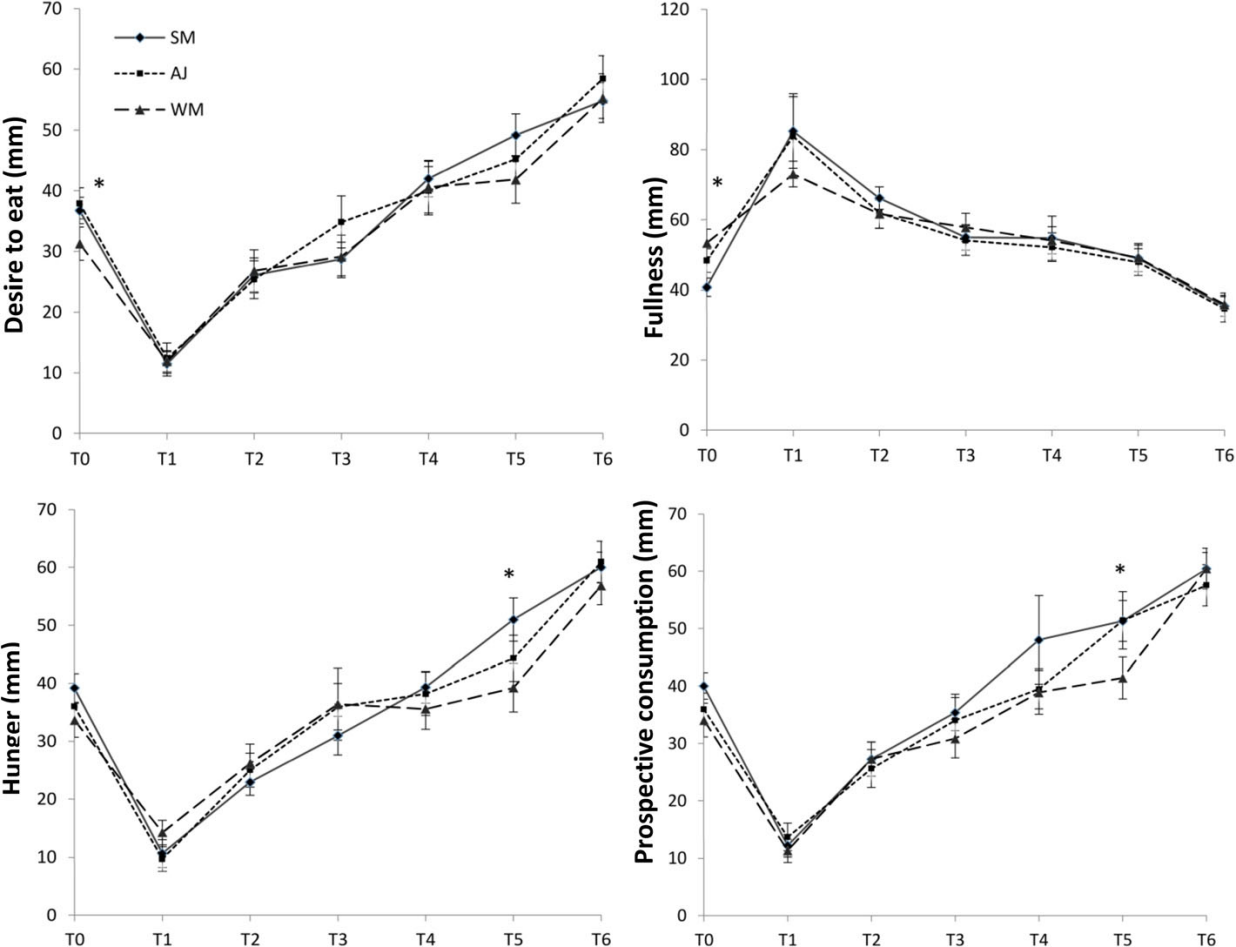
Mean ± SE subjective visual analogue scale (VAS) values for desire to eat, fullness, hunger and prospective consumption after drinking the skim milk (●), apple juice (■) and whole milk (▲) adjusted for age, gender, BMI, and rolling method. Significant differences between the intervention periods are shown with asterisks. Standard errors are appeared by vertical bars

The comparison between the effects of intervention beverages on different appetite components in all participants are provided in Fig. 3. The desire to eat was significantly higher before consumption of the breakfast containing apple juice compared to whole milk (*P* < 0.05); furthermore, the sense of fullness was significantly higher before breakfast in the whole milk period compare to skim milk period (*P* < 0.05). It was shown that the level of Hunger and prospective consumption of food was significantly lower at 11:30 a.m. when participants consumed whole milk at breakfast compared to skim milk (*P* < 0.05).

The gender-specific effect of intervention beverages on the overall appetite and its components are provided in Additional file 1. The mean appetite score was lower right after breakfast consumption (7:30 a.m.) in male participants when they consumed apple juice with their breakfast compared to whole milk (*P* < 0.05). There was no difference between beverages in other testing occasions when the analysis was done in male participants (Additional file 1: Figure S1). The male participants felt higher levels of fullness before eating breakfast with whole milk when compared to skim milk (*P* < 0.05). However, the sense of hunger was significantly higher right after breakfast after drinking the whole milk compared to apple juice (*P* < 0.05) (Additional file 1: Figure S2).

The appetite score was significantly lower before drinking the whole milk with breakfast compared to skim milk in girls (*P* < 0.05). At 9:30 a.m. (T3) the appetite score was significantly higher after drinking the apple juice compared to skim milk in females (*P* < 0.05); furthermore, the appetite was significantly lower at 11:30 a.m. (T5) after drinking the whole milk compared to skim milk (*P* < 0.05) (Additional file 1: Figure S3). The same associations were found for the desire to eat in the female participants (Additional file 1: Figure S4). Moreover, the sense of fullness was significantly higher at 9:30 a.m. in females when the skim milk was compare to apple juice (*P* < 0.05). It is also revealed that the sense of prospective consumption was significantly higher after drinking the skim milk and apple juice compared to whole milk (*P* < 0.05) (Additional file 1: Figure S4).

## Discussion

The present three-way randomized cross-over trial among children revealed that the whole milk, skim milk and apple juice does not differently affect the energy intake at lunch in either gender as well as the total population. Although the test beverages were not different in their effect on short-term energy intake, it was shown that drinking the whole milk decreases the appetite, sense of hunger and prospective consumption of food four hours after breakfast at 11:30 a.m. compared to skim milk. This finding was true in female participants but not in males. Furthermore, the appetite and sense of desire to eat were significantly lower in girls two hours (9:30 a.m.) after drinking the skim milk compared to apple juice. Previous studies have examined the effect of dairy consumption on satiety and short-term energy intake [17]. However, the different effect of full-fat milk compared to skim milk on the energy intake is not well understood. We are not aware of previous studies trying to compare dairy foods with the different fat content on the energy intake in the subsequent meal and the satiety.

The present study revealed that skim milk significantly lowers the appetite score compared to apple juice about two hours after preloads in female children but not in boys. In a study conducted by Dove et al., the consumption of skim milk, in comparison with a fruit drink, led to increased perceptions of satiety and a decreased energy intake at a subsequent meal [6]. The same effect was also shown in a study done by Mehrabani et al. [7] in which they found that low-fat milk significantly reduces the appetite and modestly decreases the short term energy intake compared to apple juice. However, the total whole day energy intake was not significantly different between the three interventions in another report by this team [18].

In the present study, we could not see the energy intake lowering effect of whole and skim milk compared to apple juice. In fact, Mehrabani et al. explored the effect of low fat milk compared to apple juice. However, the in the present study skim milk and whole milk were compared to apple juice.

Several mechanisms are suggested for the appetite lowering effect of milk intake when compared to fruit juice. Milk might exert its effect on satiety because of its high content of protein. It has been shown that proteins are more satiating in comparison with carbohydrates [19]. Changes in appetite-linked hormones, amino acids, and gluconeogenesis might be after protein consumption responsible for such an effect [20]. It is not clear that to which extent whey or casein proteins, exclusively or mixed might affect appetite. Both proteins are associated with this effect [20]. Milk also is high in lactose which is associated with reduced appetite, too. The satiating effect of lactose on energy intake, was reported to be the same as dairy protein [21]; both preloads significantly reduced the energy intake when compared with a glucose containing preload. It is also revealed that viscous beverages might have appetite lowering effect and decrease the energy intake [22]. Milk is more viscous compared with fruit drink and this may contribute to the appetite lowering effect of this beverage. Calcium which is high in dairy products might also influence appetite [23]. However, animal studies does not support this hypothesis up to now [24].

In the present study, we showed that full-fat milk significantly reduces the satiety 4 h after its drinking when compared with skim milk. The fat component of dairy products is high in conjugated linoleic acid (CLA) and medium chain triglycerides (MCTs) which their individual effect on the energy intake and subjective appetite have been proposed by a number of studies [25–27]. A study also represented that full-fat milk delays the gastric-emptying time in comparison with half-skimmed milk [28]. However, to the best of our knowledge, no study has tried to compare the skim milk and whole milk in this regard. A recent study done by Kling et al. [29] revealed that including full-fat milk in lunch decreases the amount of the lunch eaten by the children when compared with low-fat milk but the energy intake was not significantly different between the two beverages. In fact, they did not compare the effect of milk with different fat content on the satiety and energy intake in the subsequent meal. In the present study, also we found that there was no difference between skim milk and whole milk in terms of their effect on short-term energy intake; however, satiety was significantly higher four hours after consumption of whole milk compared to skim milk. In line with our result, a previous research proposed that a 4 h interval between drinking of the beverages and initiation of a test meal may reveal the effect of milk on appetite and energy intake [6]. This may explain why this effect was not significant at the other time points in the present study.

It should be noted that only children with obesity were included in the present study and the results may be different in normal weight children. Furthermore, the subsequent meal was provided five hours after drinking the test beverages and the effect may have been different if the meal was provided earlier after the intake of preloads.

## Conclusions

In conclusion the current study revealed that providing one cup of full-fat milk, skim milk or apple juice in breakfast does not make any difference in energy intake at lunch; however, whole milk significantly reduced appetite when compared with skim milk 4 h after drinking the test beverages; which highlights the appetite-reducing effect of dairy fat. Further studies comparing the effect of full-fat versus skim milk in children exploring the effect in longer periods of time and also including normal weight participants are highly recommended.

## Additional file

Additional file 1: **Table S1.** Food and Nutrient content of the test beverages along with the fixed content breakfast. **Figure S1.** Mean ± SE subjective visual analogue scale (VAS) values for the overall appetite in male participants after intake of skim milk (●), apple juice (■) and whole milk (▲) adjusted for age, BMI and rolling method. Significant differences between the intervention periods are shown with asterisks. Standard errors are appeared by vertical bars. **Figure S2.** Mean ± SE subjective visual analogue scale (VAS) values for desire to eat, fullness, hunger and prospective consumption in male participants after ingestion of skim milk (●), apple juice (■) and whole milk (▲) adjusted for age, BMI and rolling method. Significant differences between the intervention periods are shown with asterisks. Standard errors are appeared by vertical bars. **Figure S3.** Mean ± SE subjective visual analogue scale (VAS) values for the overall appetite in female participants after intake of skim milk (●), apple juice (■) and whole milk (▲) adjusted for age, BMI and rolling method. Significant differences between the intervention periods are shown with asterisks. Standard errors are appeared by vertical bars. **Figure S4.** Mean ± SE subjective visual analogue scale (VAS) values for desire to eat, fullness, hunger and prospective consumption in female participants after ingestion of skim milk (●), apple juice (■) and whole milk (▲) adjusted for age, BMI and rolling method. Significant differences between the intervention periods are shown with asterisks. Standard errors are appeared by vertical bars. (DOCX 1706 kb)
