# Supplementary material for: The effects of whole milk compared to skim milk and apple juice consumption in breakfast on appetite and energy intake in obese children: a three-way randomized crossover clinical trial
Source: BMC Nutr. 2018 Dec 10;4:44. doi: 10.1186/s40795-018-0253-8 (PMC7050899; doi:10.1186/s40795-018-0253-8)

**Additional file 1: Table S1- Food and Nutrient content of the test beverages along with the fixed content breakfast**

|  | Skim milk (SM) | Apple juice (AJ) | Whole milk (WM) |
| --- | --- | --- | --- |
| **Ingredient** |  |  |  |
| Skim milk (ml) | 240 | - | - |
| Apple juice (ml) | - | 240 | - |
| Whole milk (ml) | - | - | 240 |
| Iranian whole wheat bread (g) | 65 | 65 | 65 |
| Walnut (g) | 12 | 12 | 12 |
| Low fat cheese (g) | 15 | 15 | 15 |
| **Nutrients** |  |  |  |
| Energy (Kcal) | 383.2 | 411.44 | 447.7 |
| Protein (g) | 19.28 | 11.276 | 18.96 |
| Fat (g) | 13.22 | 13.022 | 20.93 |
| Carbohydrates (g) | 49.09 | 65.195 | 48.59 |
| Fiber (g) | 1.04 | 1.016 | 1.04 |
|  |  |  |  |
| Glycemic index | 40.17 | 42.5 | 40.17 |
| Glycemic load | 19.70 | 27.70 | 19.70 |

**Additional file 1: Figure S1-** Mean ± SE subjective visual analogue scale (VAS) values for the overall appetite in male participants after intake of skim milk (●), apple juice (■) and whole milk (▲) adjusted for age, BMI and rolling method. Significant differences between the intervention periods are shown with asterisks. Standard errors are appeared by vertical bars.


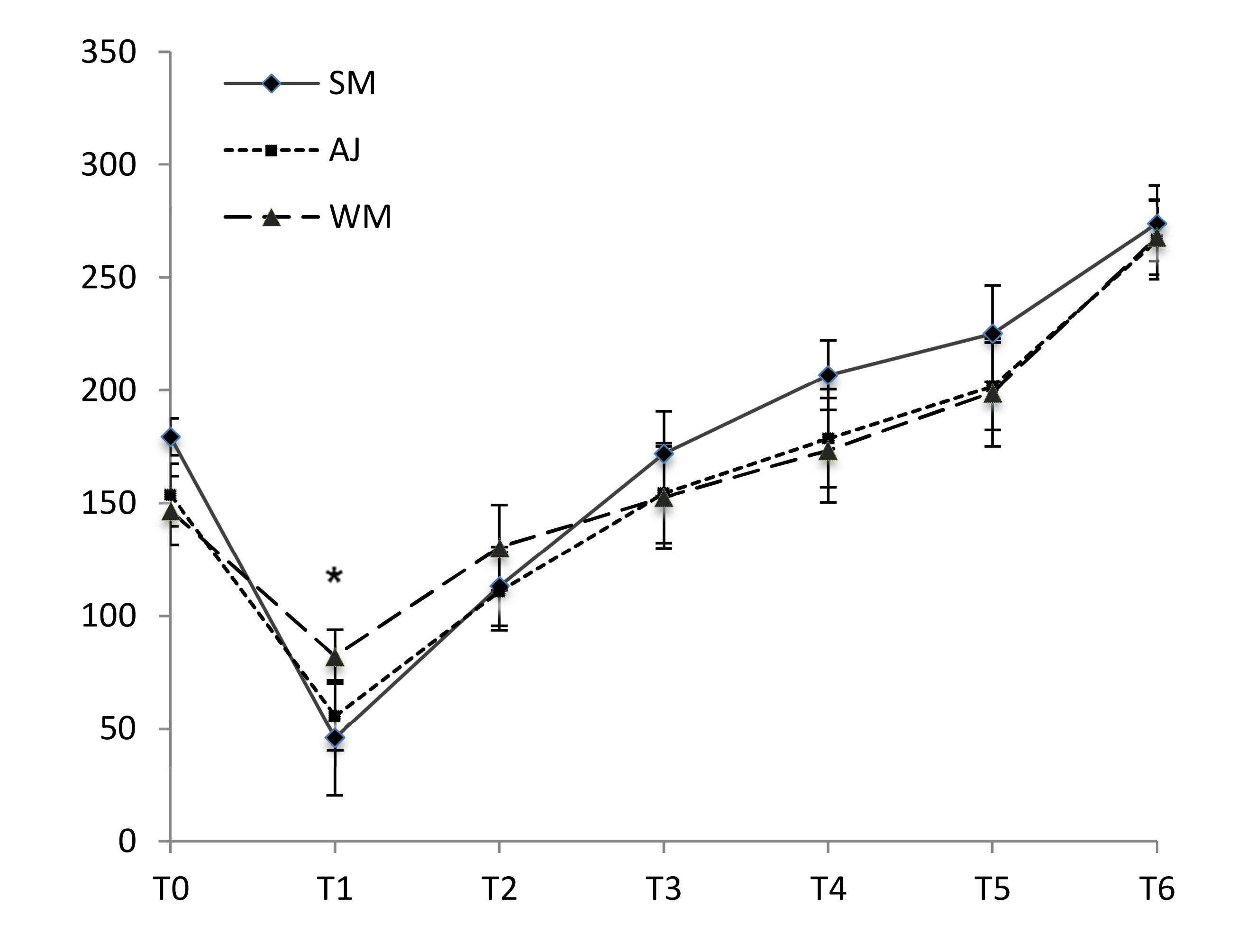


**Additional file 1: Figure S2-** Mean ± SE subjective visual analogue scale (VAS) values for desire to eat, fullness, hunger and prospective consumption in male participants after ingestion of skim milk (●), apple juice (■) and whole milk (▲) adjusted for age, BMI and rolling method. Significant differences between the intervention periods are shown with asterisks. Standard errors are appeared by vertical bars.


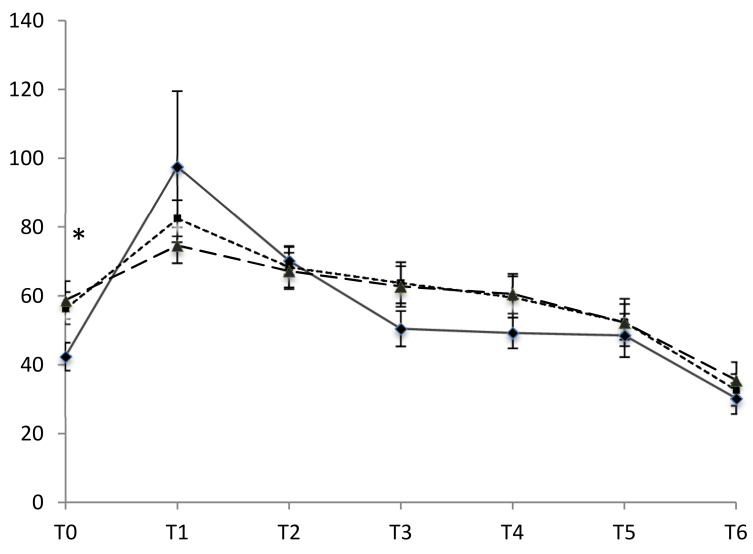


**Hunger (mm)**


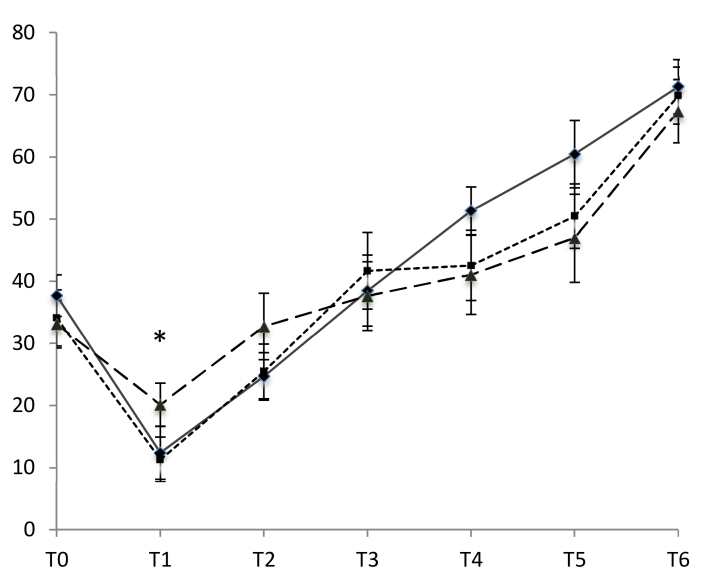


**Desire to eat (mm)**

**Prospective consumption (mm)**


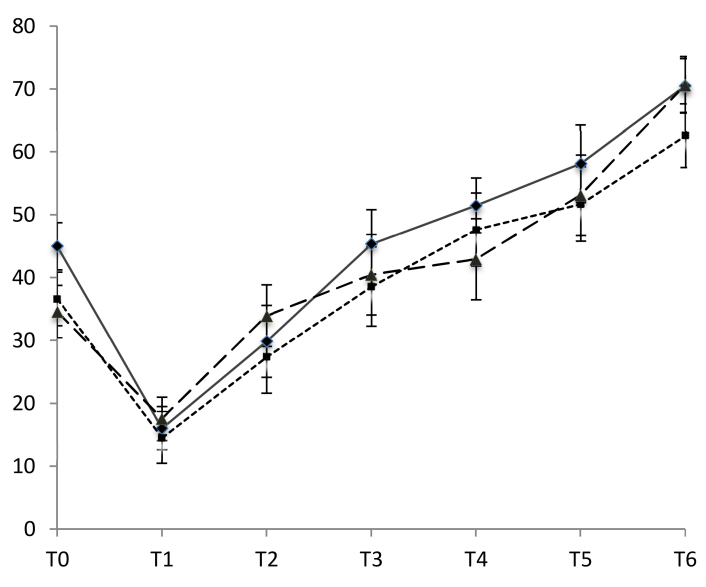


**Fullness (mm)**


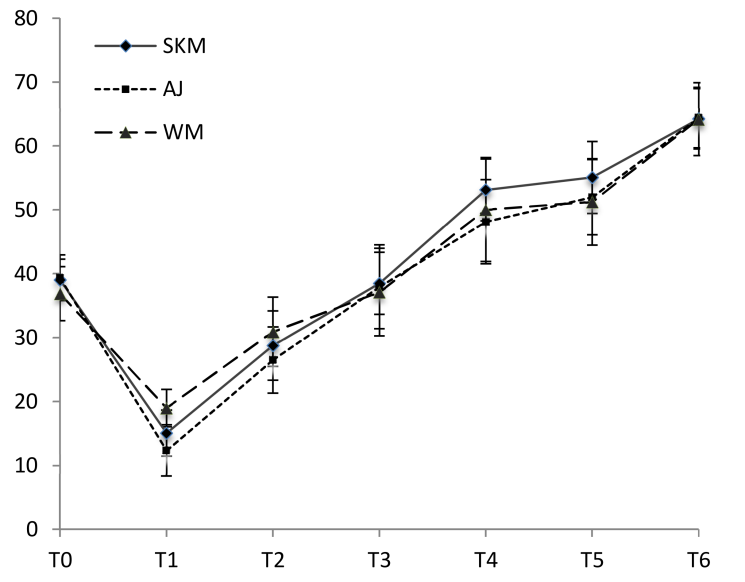


**Additional file 1: Figure S3-** Mean ± SE subjective visual analogue scale (VAS) values for the overall appetite in female participants after intake of skim milk (●), apple juice (■) and whole milk (▲) adjusted for age, BMI and rolling method. Significant differences between the intervention periods are shown with asterisks. Standard errors are appeared by vertical bars.


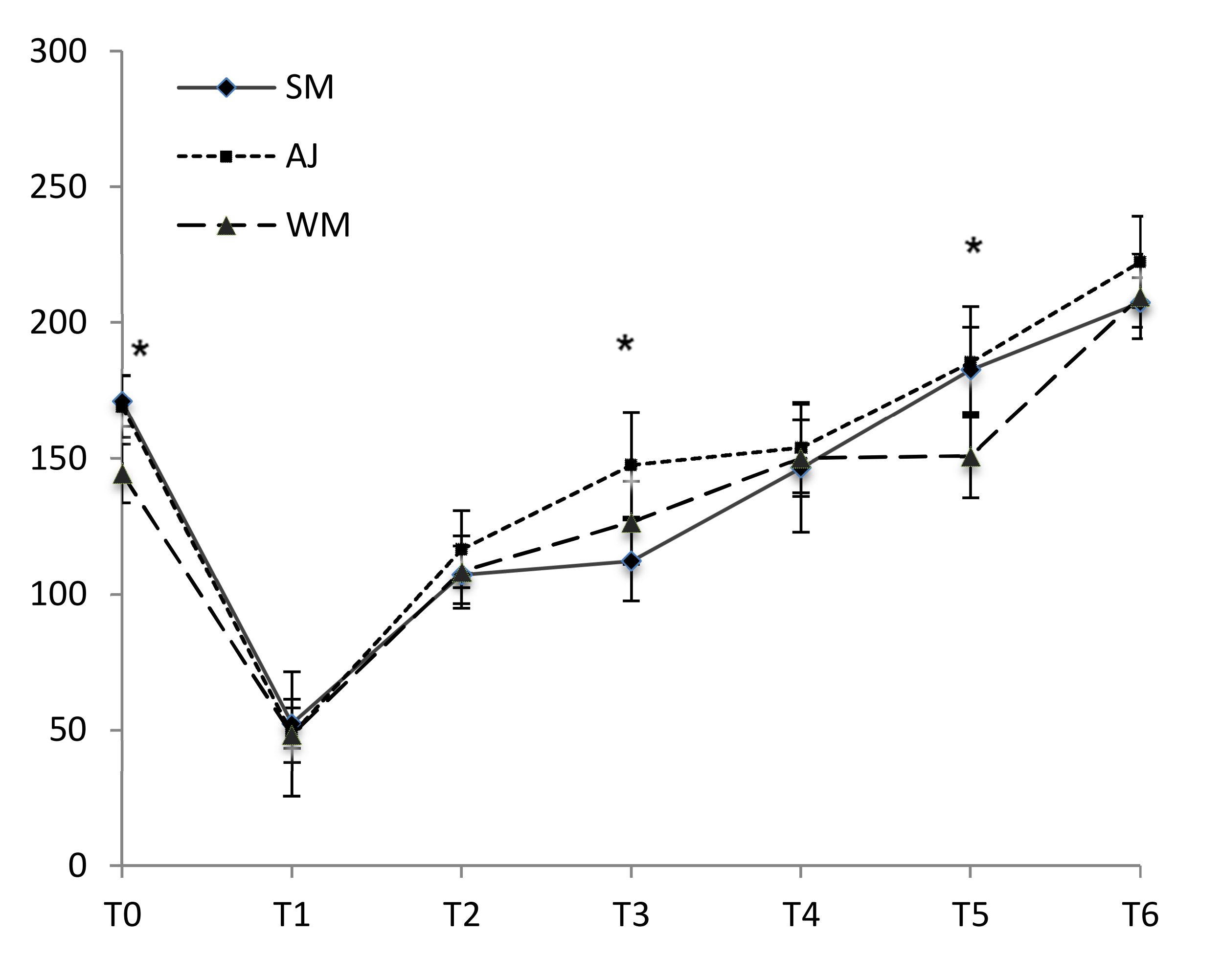


**Additional file 1: Figure S4-** Mean ± SE subjective visual analogue scale (VAS) values for desire to eat, fullness, hunger and prospective consumption in female participants after ingestion of skim milk (●), apple juice (■) and whole milk (▲) adjusted for age, BMI and rolling method. Significant differences between the intervention periods are shown with asterisks. Standard errors are appeared by vertical bars.

**Hunger (mm)**

**Fullness (mm)**

**Prospective consumption (mm)**

**Desire to eat (mm)**


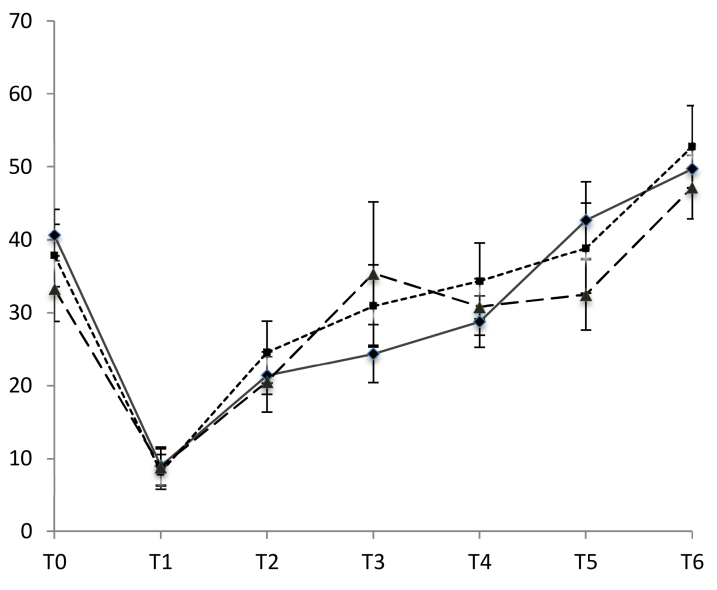

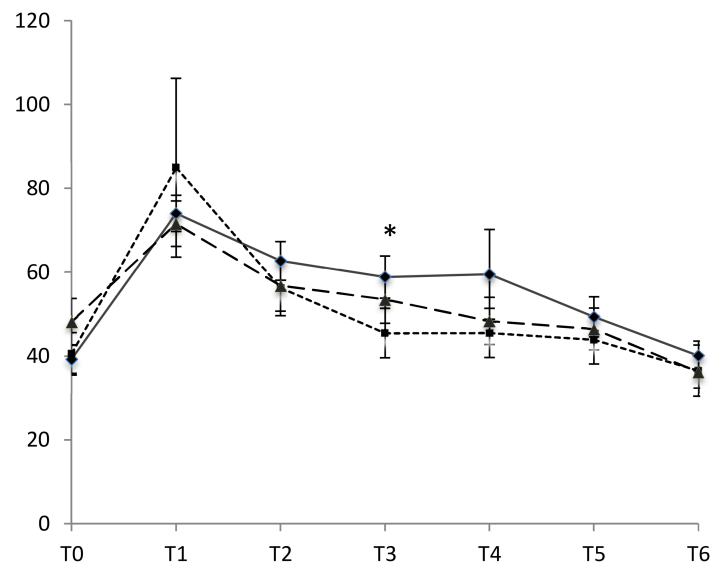

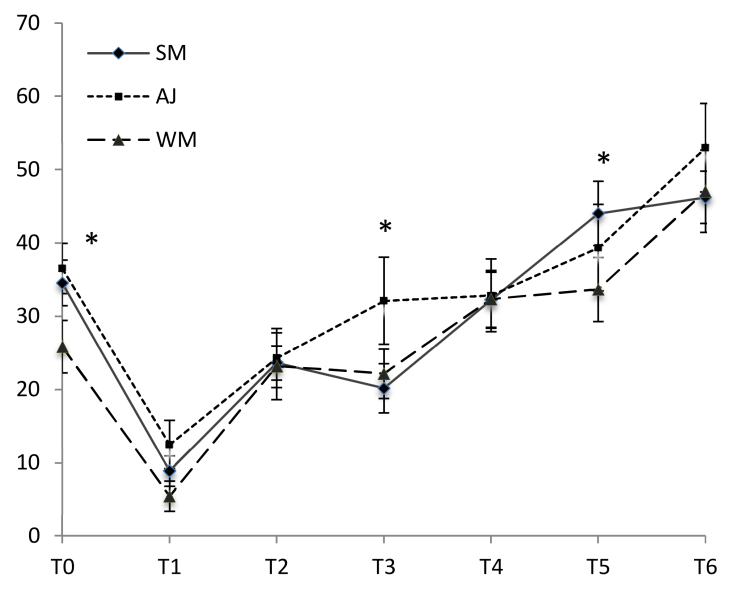

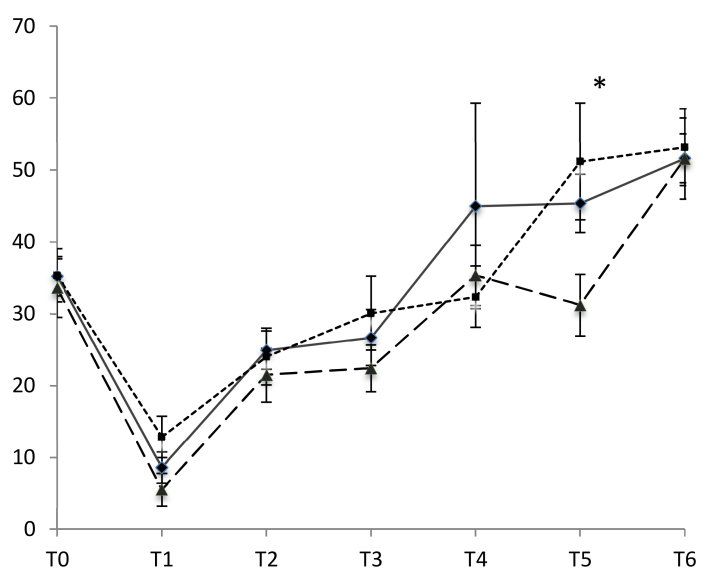

Supplement: Supplementary file 1 — Table S1. Food and Nutrient content of the test beverages along with the fixed content breakfast. Figure S1. Mean ± SE subjective visual analogue scale (VAS) values for the overall appetite in male participants after intake of skim milk (●), apple juice (■) and whole milk (▲) adjusted for age, BMI and rolling method. Significant differences between the intervention periods are shown with asterisks. Standard errors are appeared by vertical bars. Figure S2. Mean ± SE subjective visual analogue scale (VAS) values for desire to eat, fullness, hunger and prospective consumption in male participants after ingestion of skim milk (●), apple juice (■) and whole milk (▲) adjusted for age, BMI and rolling method. Significant differences between the intervention periods are shown with asterisks. Standard errors are appeared by vertical bars. Figure S3. Mean ± SE subjective visual analogue scale (VAS) values for the overall appetite in female participants after intake of skim milk (●), apple juice (■) and whole milk (▲) adjusted for age, BMI and rolling method. Significant differences between the intervention periods are shown with asterisks. Standard errors are appeared by vertical bars. Figure S4. Mean ± SE subjective visual analogue scale (VAS) values for desire to eat, fullness, hunger and prospective consumption in female participants after ingestion of skim milk (●), apple juice (■) and whole milk (▲) adjusted for age, BMI and rolling method. Significant differences between the intervention periods are shown with asterisks. Standard errors are appeared by vertical bars. (DOCX 1706 kb) [file 40795_2018_253_MOESM1_ESM.docx]
